# Supplementary material for: Is the Stalk of the SARS-CoV-2 Spike Protein Druggable?
Source: Viruses. 2022 Dec 14;14(12):2789. doi: 10.3390/v14122789 (PMC9786045; doi:10.3390/v14122789)
Supplement: Supplementary file 1 [file viruses-14-02789-s001.zip › HTVS_Supplementary_14122022.pdf]

SUPPLEMENTARY INFORMATION

Is the Stalk of the SARS-CoV-2 Spike Protein Druggable?

Ludovico Pipitò, Christopher A. Reynolds, and Giuseppe Deganutti

Centre for Sport, Exercise and Life Sciences, Faculty of Health and Life Sciences, Coventry University, Coventry CV1 5FB, UK

Video S1. Fragment 3 bound to sub-pocket 2 (binding mode 1). One molecule of fragment 3 is depicted as in green stick, the SP stalk as a white transparent ribbon, and glycan residues as black lines; hydrogen bonds are shown as red dashed lines.

Video S2. Fragment 3 bound to sub-pocket 2 (binding mode 2). Three molecules of fragment 3 occupy a sub-pocket 2 each on the surface of the SP stalk. Fragment 3 is depicted as in green stick, the SP stalk as a white transparent ribbon, and glycan residues as black lines; hydrogen bonds are shown as red dashed lines

Table S1. SMILES of the fragments used.

Table S2. Fragments' docking poses, ranked according to the docking score.

Table S3. DES of the 559 fragments. The average (avg) and standard deviation (SD) of DES, GBSA, and RMSD are also reported.

Table S4. The best 18 fragments according to post-docking MD.

Table S5. The best 3 fragments (1-pyridin-2-ylpiperidin-4-one, N-phenyl-1,3,4-thiadiazol-2-amine, 6-fluoro-3-piperidin-4-yl-1H-indole); their MIxMD contacts' occupancies are indicated individually with the respective frequency expressed in % over the totality of the MD frames.

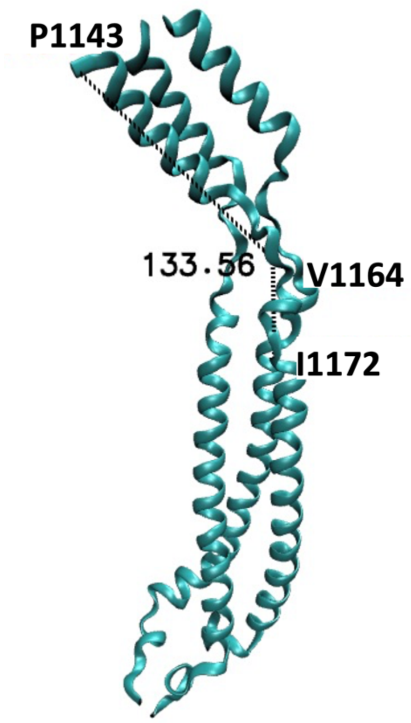

Figure S1. Our simulation indicated that an angle of 133.56° is formed between P1143, V1164, and I1172 causing important changes in the structure with the consequent opening of broad gaps between the chains with P1143 and G1171 being key residues for structural flexibility.

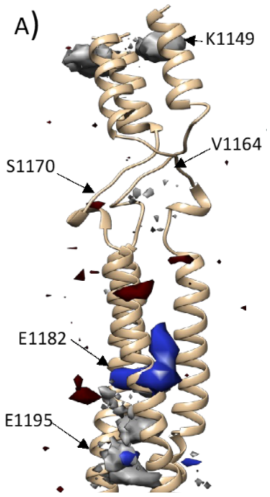

Figure S2. MixMD Density maps for BENZ (grey), FAC (blue), and MTA (red) at the 20% iso value. The probes identified possible sites on the stalk (ribbon) accessible to small molecules, despite the presence of glycans (not shown for clarity).

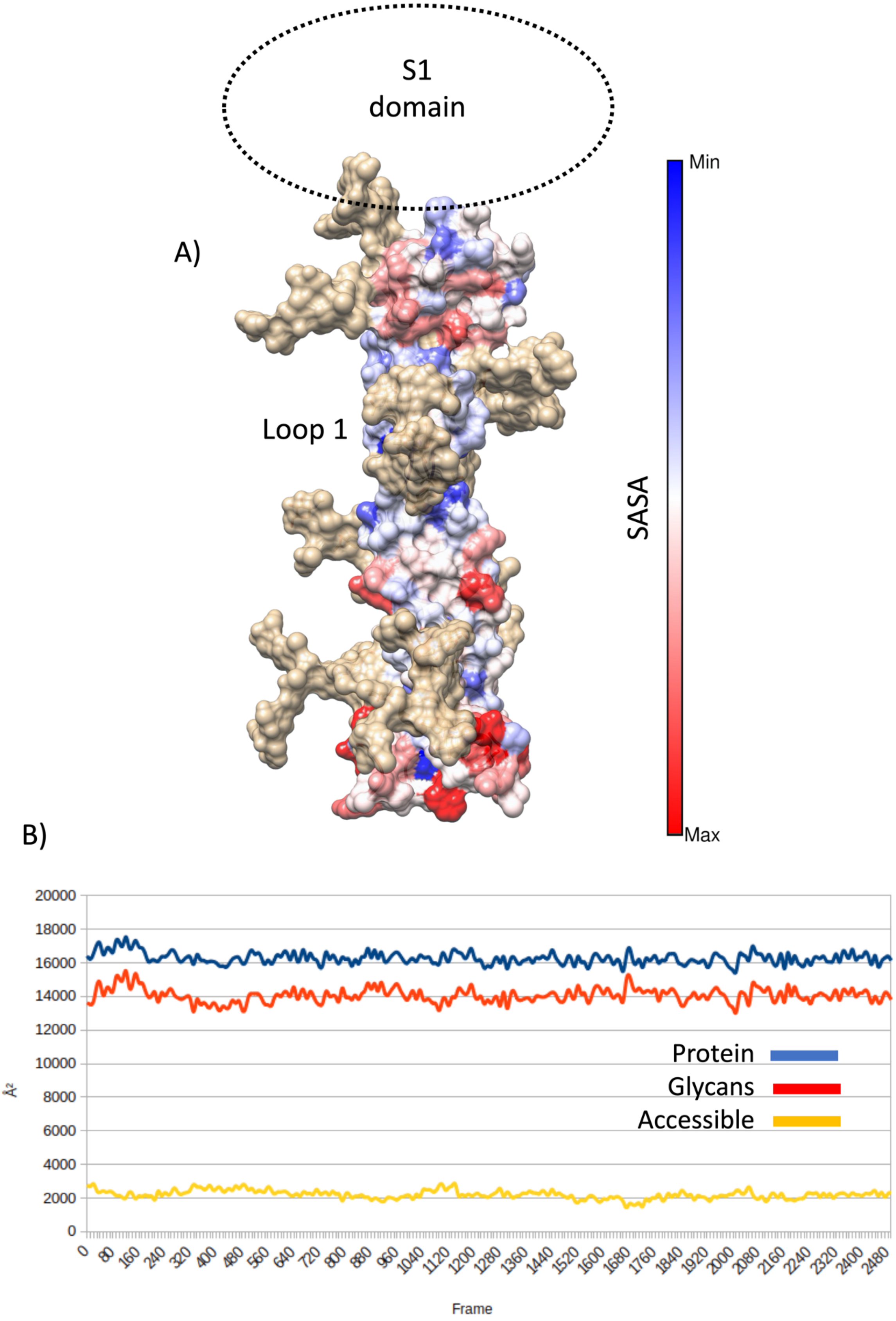

Figure S3. A) Solvent Accessible Surface Area (SASA) indicates possible accessible sites (red color) toward the N-terminal of the stalk, below the connection with S1, and below Loop 1;

glycans are shown as tan surface. **B**) Glycan residues movement tends to give access to a small portion of the stalk and has a considerable impact on the accessible surface.

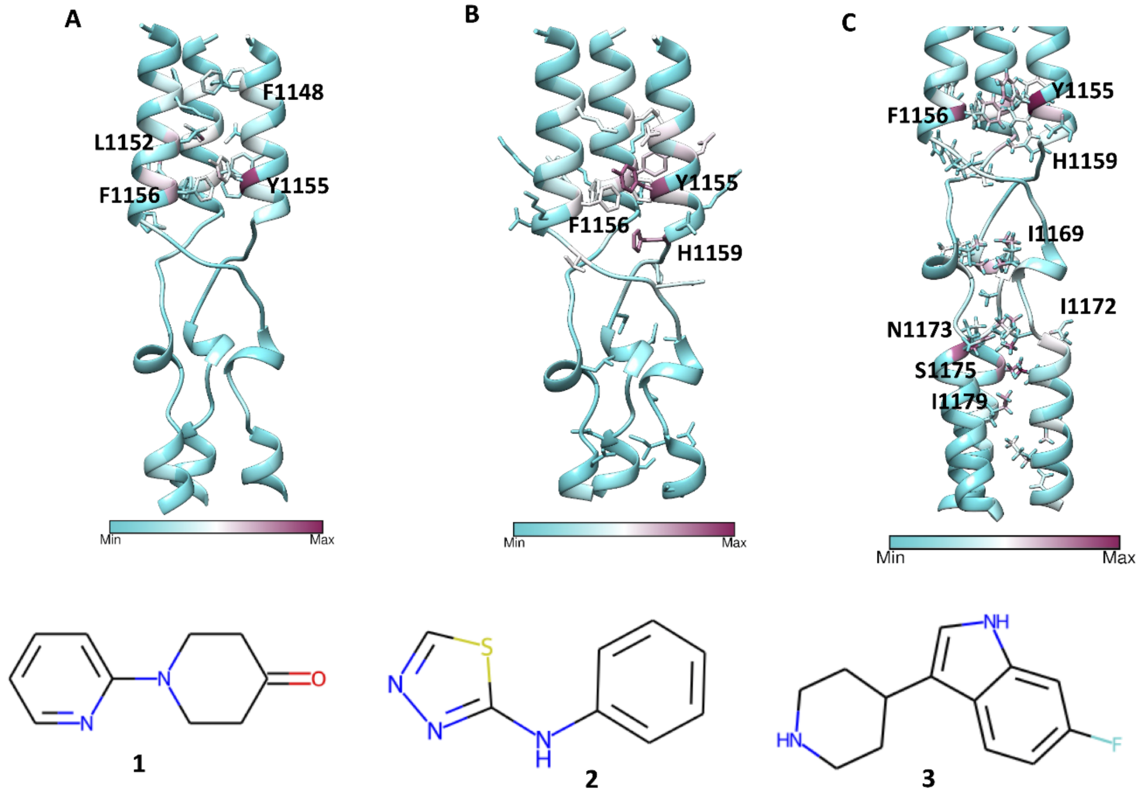

Figure S4. Contacts formed during mixMD simulations of fragments 1-3. The stalk is reported as a cyan ribbon, while the most involved residues (**Table S5**) are in stick maroon representation.
